# Citation Velocity and Social Media Impact Analysis of Hip and Knee Arthroplasty Randomized Controlled Trials

**DOI:** 10.1016/j.artd.2026.102046

**Published:** 2026-05-17

**Authors:** James R. Temple, Nadim Barakat, Logan S. Carpenter, Wendy M. Novicoff, James A. Browne

**Affiliations:** a Department of Orthopaedic Surgery, University of Virginia, Charlottesville, VA, USA; b Department of Orthopedic Surgery, Virginia Commonwealth University, Richmond, VA, USA; c Department of Public Health Sciences and Orthopaedic Surgery, University of Virginia, Charlottesville, VA, USA

**Keywords:** Arthroplasty research, Impact, Bibliometric analysis, Randomized controlled trials

## Abstract

**Background:**

Randomized controlled trials (RCTs) remain crucial in establishing evidence-based care, yet little is known about which studies drive the most academic and public attention. This study aimed to identify the most impactful hip and knee arthroplasty RCTs published from 2014 through 2023 using traditional citation metrics and Altmetric data as well as determining study characteristics associated with greater impact.

**Methods:**

Clinically oriented hip and knee arthroplasty RCTs were identified and analyzed from 4 leading orthopedic journals published from 2014 through 2023. Article impact was assessed by citation velocity (citations per year) and Altmetric Attention Scores (AAS). Inequality in citation and AAS distributions was measured using Gini coefficients. Mann-Whitney U tests were used to explore associations between impact metrics and study characteristics such as funding and the presence of statistically significant findings.

**Results:**

Among the 566 RCTs, the mean citation velocity was 5.3 citations per year, and the mean AAS was 12.3. Citation velocity and AAS were weakly correlated (r = 0.34, *P* < .01). Gini coefficients were 0.42 and 0.77 for citation velocity and AAS, respectively, indicating a highly unequal distribution of both scholarly and online attention. Industry funding was not associated with citation velocity (*P* = .988) or AAS (*P* = .957). However, studies with statistically significant results in the primary outcome favoring the experimental intervention had an 18% higher citation velocity than those without (*P* = .018).

**Conclusions:**

The impact of hip and knee arthroplasty RCTs is highly skewed, with a small number of articles receiving a disproportionate amount of attention. Importantly, RCTs with statistically significant results were more likely to be cited, suggesting that favorable findings may disproportionately shape the arthroplasty literature.

## Introduction

Randomized controlled trials (RCTs) serve as the gold standard for clinical research and are foundational to evidence-based decision-making in orthopedic surgery [1]. In the field of hip and knee arthroplasty, RCTs are crucial for evaluation of new technologies, implants, surgical techniques, pain management strategies, and more [2]. While the volume of published research continues to grow, not all trials have exerted similar influence. It is important to determine which trials have received the most attention to understand trends in arthroplasty research and to determine if certain study characteristics may influence how widely a trial is disseminated and cited, which may impact its integration into clinical practice and patient care.

Traditionally, academic impact has been measured through citation counts, which reflect how often a study is referenced in subsequent scholarly work [[3], [4], [5]]. More recently, the Altmetric Attention Score (AAS) has emerged as a complementary metric, measuring public and professional interest across online platforms [[6], [7], [8], [9], [10], [11]]. The purpose of this study was to identify the most impactful hip and knee arthroplasty RCTs published between 2014 and 2023 in 4 leading orthopedic journals. Both traditional citation and AAS metrics were used to quantify article impact and attention, and this study also sought to identify if certain article characteristics such as funding source were associated with greater impact.

## Material and methods

### Sampling and inclusion criteria

PubMed was queried in May 2024 to identify articles published between January 1, 2014, and January 1, 2024, in 4 leading orthopedic journals publishing arthroplasty research: *The Bone & Joint Journal*, *Clinical Orthopaedics and Related Research*, *The Journal of Bone and Joint Surgery*, and the *Journal of Arthroplasty*. Consistent with prior bibliometric analyses that have employed targeted search strategies focused on high-impact journals [10,12,13], these 4 journals were selected because they represent the highest journal impact factor scores among orthopedic journals that routinely publish clinically oriented hip and knee arthroplasty RCTs. Additionally, restricting the analysis to journals with comparable journal impact factors was intended to limit potential confounding related to journal visibility and impact scores.

**Figure 1:**
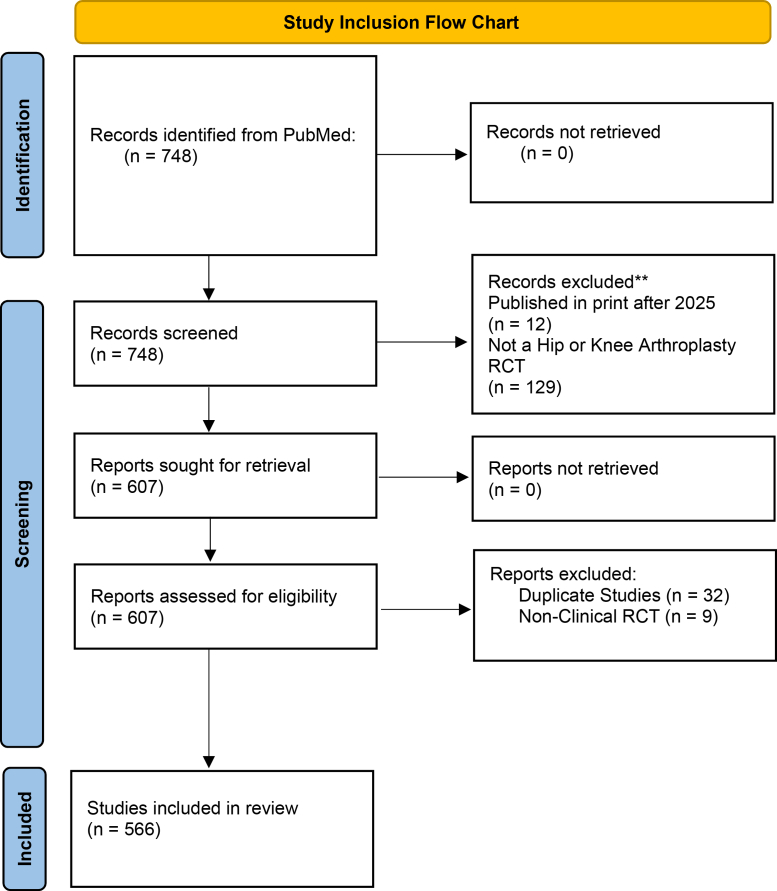
Study inclusion flow chart.

Using automatic term mapping and MeSH terms, the search string for each journal included: “hip arthroplasty OR knee arthroplasty” AND “randomized controlled trial” (Fig. 1). Eligible publications were prospective, randomized controlled studies in hip and/or knee arthroplasty that were clinically focused and compared 2 or more interventions across multiple groups. Exclusion criteria were non-RCT study designs, duplicate publications, or articles unrelated to patient care or hip and knee arthroplasty. When a duplicate represented a follow-up to an original RCT, only the initial publication was included. Abstracts were screened by a single reviewer to assess eligibility, followed by independent full-text review by 2 reviewers to determine the final study cohort (Fig. 1).

### Data collection

The following data were extracted from each article: journal of publication, joint studied, country of origin, funding source, experimental intervention category, and study result. Interventions were broadly categorized into 4 categories based on the topic of the research study: surgical technology, surgical procedures/technique, pain and perioperative management, and outpatient centered care (see Table 1 for examples of each category). Funding source was dichotomously classified into industry or not based on funding statements associated with the articles. Study result was dichotomously classified as either favorable or not favorable based on a statistically significant result of the primary outcome. For example, if the experimental group demonstrated a statistically significant and superior performance compared to the control group, this was considered a “favorable” result. If there was no statistically significant difference between the experimental treatment and the control, or if the experimental arm performed worse than the control, the result was considered “not favorable.”

**Table 1:** Examples of intervention category classification.

Surgical technology	Surgical procedures and technique	Pain and perioperative management	Outpatient centered care
Robotic-assisted unicompartmental knee arthroplasty	Fracture Fixation vs Arthroplasty for displaced femoral neck fractures	Femoral Nerve Block vs Periarticular Infiltration	Self-directed Home Exercise Program
Fixed and mobile-bearing implants	Kinematic vs Mechanical Alignment	Intravenous and Periarticular Corticosteroids	In-home Telerehabilitation Program
Patient-specific cutting guides	Direct lateral vs Anterolateral Approach	Methods of Tranexamic Acid Administration	Smartphone-based care platform

An article’s impact and attention were evaluated using both the Altmetric Attention Score and citation data. The Altmetric Attention Score (AAS) is a single quantitative measure that captures the online attention an article receives. It is calculated using a weighted algorithm that assigns different values to mentions from various platforms, such as news outlets, blogs, X/Twitter, Wikipedia, and policy documents [14]. A higher numerical AAS reflects greater online attention. Several bibliometric studies have used AAS to measure impact within orthopedic literature [[15], [16], [17]].

Citation data were collected using the SCOPUS database (Elsevier, 2024), a well validated tool for citation data collection used in bibliometric analyses [18]. AAS was collected through the Altmetric Bookmarklet application. An article’s AAS and citation count are dynamic metrics that change over time. To reduce time bias, all impact data were collected in a single 3-day window, from March 3, 2025, to March 6, 2025.

In addition, because articles published earlier in the sample would have had more time to accrue citations, citation count was normalized by using citation velocity. Citation velocity was calculated by the number of citations divided by the years in circulation (2025 minus publication year). For example, if an article published in 2020 had 50 citations, its citation velocity would be normalized to 10 citations/year (50 citations/5 years in circulation). AAS was analyzed as raw data, not normalized in this fashion. AAS was not normalized based on prior research indicating that AAS does not increase linearly with time like citations but reflects the immediate influence of an article shortly after the time of publication [11,19]. For example, it is unlikely for articles that have been already published for several years to suddenly get posted on social media websites compared to how citations accrue over time.

### Data analyses

Data were analyzed using both descriptive statistics and comparative statistics. Descriptive statistics included mean and standard deviation impact data, distribution of articles by intervention category, correlation between AAS and citation velocity, and calculating the Gini coefficient for citation velocity and AAS. The Z-score for all articles was calculated for citation velocity and AAS in order to isolate outliers with the highest scores for more focused review. Articles with a Z-score >3.00 were included in Table 2 and Table 3 to feature the most impactful RCTs from 2014-2023.

**Table 2:** Articles with the highest altmetric attention scores.

Title	Altmetric score	Journal	Country	Year	Joint	Exact intervention	Intervention category	Primary outcome result
Improved Accuracy of Component Positioning with Robotic-Assisted Unicompartmental Knee Arthroplasty: Data from a Prospective, Randomized Controlled Study	535	JBJS	United Kingdom	2016	Knee	Robotic-assisted unicompartmental knee arthroplasty	Surgical technology	Favorable
Cemented vs Cementless Total Knee Arthroplasty of the Same Modern Design: A Prospective, Randomized Trial.	519	JBJS	United States	2019	Knee	Cemented vs uncemented total knee arthroplasty	Surgical procedures and techniques	Not Favorable
Intraosseous Morphine Decreases Postoperative Pain and Pain Medication Use in Total Knee Arthroplasty: A Double-Blind, Randomized Controlled Trial.	464	JOA	United States	2022	Knee	Intraosseous morphine	Pain and perioperative management	Favorable
Comparison of Functional Recovery Between Unicompartmental and Total Knee Arthroplasty: A Randomized Controlled Trial.	296	JBJS	Thailand	2023	Knee	Unicompartmental knee arthroplasty	Surgical procedures and techniques	Favorable
A pragmatic randomised controlled trial comparing the efficacy of a femoral nerve block and periarticular infiltration for early pain relief following total knee arthroplasty.	196	BJJ	United Kingdom	2017	Knee	Femoral nerve block vs periarticular infiltration	Pain and perioperative management	Not favorable
Hemiarthroplasty or total hip arthroplasty for the treatment of a displaced intracapsular fracture in active elderly patients: 12-year follow-up of randomised trial.	196	BJJ	The Netherlands	2017	Hip	Cemented hemiarthroplasty vs cemented total hip arthroplasty	Surgical Procedures and Techniques	Not favorable
Local Infiltration Analgesia With Liposomal Bupivacaine Improves Pain Scores and Reduces Opioid Use After Total Knee Arthroplasty: Results of a Randomized Controlled Trial.	188	JOA	United States	2018	Knee	Liposomal Bupivacaine	Pain and Perioperative Management	Favorable
A randomized controlled trial comparing the Thompson hemiarthroplasty with the Exeter polished tapered stem and Unitrax modular head in the treatment of displaced intracapsular fractures of the hip	179	BJJ	United Kingdom	2018	Hip	Traditional cemented monoblock Thompson hemiarthroplasty vs modern cemented modular polished-taper stemmed hemiarthroplasty	Surgical Technology	Not favorable

JOA, Journal of Arthroplasty; JBJS, Journal of Bone and Joint Surgery; BJJ, Bone & Joint Journal.

**Table 3:** Articles with the greatest citation velocity.

Title	Citation velocity	Journal	Country	Year	Joint	Exact intervention	Intervention category	Conclusion
Effects of Virtual Exercise Rehabilitation In-Home Therapy Compared with Traditional Care After Total Knee Arthroplasty	30	JBJS	United States	2020	Knee	Virtual rehab	Outpatient-centered care	Favorable
A randomised controlled trial of kinematically and mechanically aligned total knee replacements: 2-year clinical results.	29.4	BJJ	United States	2014	Knee	Kinematically aligned total knee arthroplasty	Surgical procedures and techniques	Favorable
Does Robotic-assisted TKA Result in Better Outcome Scores or Long-Term Survivorship Than Conventional TKA?	29	CORR	South Korea	2020	Knee	Robotic-assisted total knee arthroplasty	Surgical technology	Not favorable
Restoring the constitutional alignment with a restrictive kinematic protocol improves quantitative soft-tissue balance in total knee arthroplasty: a randomized controlled trial.	26.2	BJJ	Australia	2020	Knee	Kinematic vs mechanical alignment	Surgical procedures and techniques	Favorable
Otto Aufranc Award: A Multicenter, Randomized Study of Outpatient vs Inpatient Total Hip Arthroplasty.	25.9	CORR	United States	2017	Hip	Same day discharge vs overnight hospital stay	Pain and perioperative management	Not favorable
Improved Accuracy of Component Positioning with Robotic-Assisted Unicompartmental Knee Arthroplasty: Data from a Prospective, Randomized Controlled Study	25.1	JBJS	United Kingdom	2016	Knee	Robotic-assisted unicompartmental knee arthroplasty	Surgical technology	Favorable
The Chitranjan S. Ranawat Award: No Difference in 2-year Functional Outcomes Using Kinematic vs Mechanical Alignment in TKA: A Randomized Controlled Clinical Trial.	23	CORR	New Zealand	2017	Knee	Kinematic vs mechanical alignment	Surgical procedures and techniques	Not favorable
In-Home Telerehabilitation Compared with Face-to-Face Rehabilitation After Total Knee Arthroplasty: A Noninferiority Randomized Controlled Trial.	20.5	JBJS	Canada	2015	Knee	In-home telerehabilitation program	Outpatient-centered care	Not favorable
Multimodal pain management in total knee arthroplasty: a prospective randomized controlled trial.	19	JOA	United States	2014	Knee	Multimodal block	Pain and perioperative management	Favorable
A Prospective Randomized Clinical Trial in Total Hip Arthroplasty-Comparing Early Results Between the Direct Anterior Approach and the Posterior Approach.	18.6	JOA	Australia	2017	Hip	Direct anterior approach vs posterior approach	Surgical procedures and techniques	Not favorable

JBJS, Journal of Bone and Joint Surgery; JOA, Journal of Arthroplasty; BJJ, Bone & Joint Journal; CORR, Clinical Orthopaedics and Related Research.

The Gini coefficient is a well-established metric in economics, often used to quantify inequality in the distribution of income or wealth within a population [20]. Its value is based on a scale between 0.0 and 1.0. A Gini score of 0 indicates perfect equality among the distribution, while a score of 1 indicates perfect inequality. In this study, the Gini coefficient, alongside Lorenz curves, was applied to assess the distribution of scholarly impact, measured by citation velocity and AAS. This approach enables determination of whether, and to what extent, a small subset of arthroplasty RCTs disproportionately accounts for the overall citations and online attention.

For univariate analyses, Mann-Whitney U and Kruskal-Wallis H tests were used to evaluate for associations between variables of interest such as citation velocity or AAS and funding source, intervention category, and study result. For multivariate analysis, linear regression analyses were also conducted to assess associations between citation velocity and AAS with predictors such as intervention category, funding source, and study result (favorable vs not favorable). Statistical significance was set to 0.05. Data analyses were conducted using IBM Statistical Package for Social Sciences (SPSS) Statistics version: 29.0.2.0 (20; Armonk, New York, USA). This study did not involve any human participants, and institutional review board approval was not needed. This study did not require any funding.

## Results

There were 566 articles included in this study. Articles were classified based on the focus of their experimental treatment as follows: outpatient centered care, 40 articles (7%); surgical procedures and technique, 126 articles (22%); surgical technology, 170 articles (30%); pain and perioperative management, 230 articles (41%).

**Figure 2:**
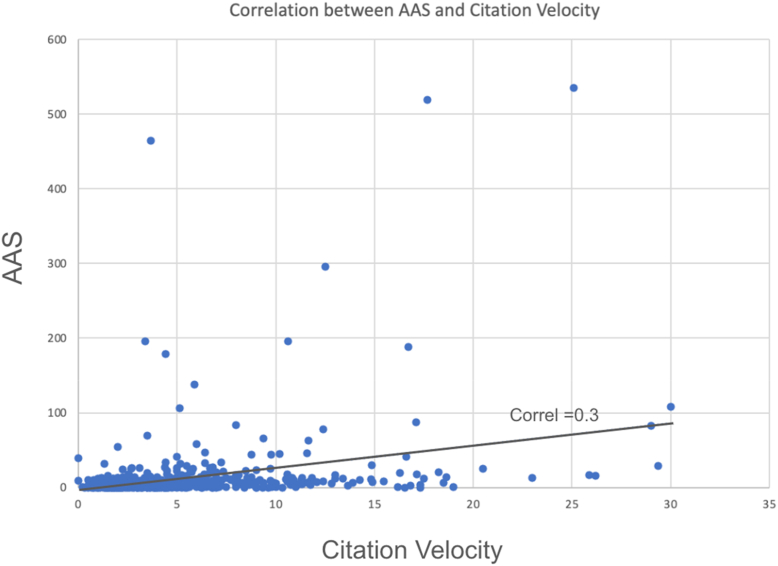
The correlation between two alternative metrics of impact: citation velocity and Altmetric Attention Score (AAS). The correlation coefficient was r = 0.30, demonstrating a weak, positive correlation.

**Figure 3:**
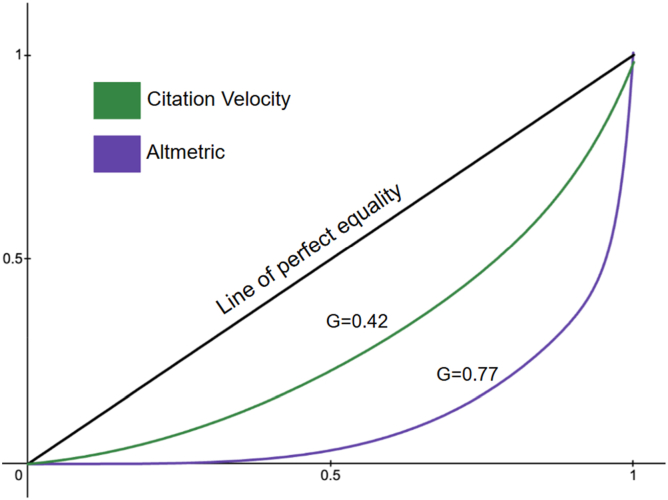
This graph depicts a Lorenz curve, which is a graphical representation of the Gini coefficient, a scale from 0.0 to 1.0, measuring distribution inequality. A Gini coefficient of 0.0 indicates perfect equality among the distribution, while a score of 1.0 indicates perfect inequality. The further a Lorenz curves moves away from the line of perfect equality, in a down and rightward fashion, the greater the measured inequality. As seen in the curves, citation velocity exhibits moderate inequality (G = 0.42), while Altmetric Attention Score (AAS) exhibits severe inequality (G = 0.77).

The mean citation velocity for all articles was 5.3 citations per year (standard deviation: 4.4) and median citation velocity was 4.0 (interquartile range: 2.4-6.5). The mean AAS for all articles was 12.3 (standard deviation of 43.4), and median AAS was 4.0 (interquartile range: 1.0-9.0). Pearson correlation coefficient between citation velocity and AAS was 0.304 (*P* < .01) revealing a weakly positive correlation (Fig. 2). For AAS, 8 RCTs were identified as having a Z-score >3.00 (Table 2) [[21], [22], [23], [24], [25], [26], [27], [28]]. For citation velocity, 10 RCTs were identified as having a Z-score >3.00 (Table 3) [21,[29], [30], [31], [32], [33], [34], [35], [36], [37]]. The Gini coefficient for the distribution of citation velocity was 0.42, indicating a moderate level of inequality. Whereas the Gini coefficient for the distribution of AAS was 0.77, indicating severe inequality (Fig. 3).

Kruskal-Wallis H tests demonstrated that the intervention category was associated with citation velocity (*P* = .028), but Dunn’s pairwise analyses were unable to identify any specific pairwise differences between only 2 categories that were statistically significant. Kruskal-Wallis H tests also demonstrated that intervention category was associated with AAS (*P* = .001). Dunn’s pairwise analyses identified that articles focused on surgical technology had significantly lower AAS compared to articles focused on pain and perioperative management (*P* = .002) and outpatient-centered care (*P* = .029) (Table 4).

**Table 4:** Impact analysis across research area of interest.

Categories of intervention	Citation velocity	AAS
Outpatient-centered care, N = 40		
Mean (95% CI)	6.6 (4.77-8.47)	15.5 (7.73-23.17)
Median (IQR)	5 (5.78)	5.5^a^ (17)
Pain and perioperative management, N = 230		
Mean (95% CI)	5.3 (4.78-5.74)	10.8 (6.14-15.47)
Median (IQR)	4.3 (4.27)	4.5^a^ (8)
Surgical procedures and techniques, N = 126		
Mean (95% CI)	5.9 (4.96-6.92)	17.6 (7.49-27.79)
Median (IQR)	4 (4.64)	3.5 (10)
Surgical technology, N = 170		
Mean (95% CI)	4.6 (3.95-5.15)	9.6 (3.02-16.27)
Median (IQR)	3.8 (3.75)	2^a^ (5)
Kruskal-Wallis, N = 566	*P* = .028	*P* = .001

CI, confidence interval; IQR, interquartile range.

a Indicates a significant difference after Dunn's pairwise adjusted significance.

Mann-Whitney U tests found no significant difference in citation velocity (*P* = .988) or AAS (*P* = .957) between articles funded by industry and nonindustry funded articles. Mann-Whitney U tests demonstrated that articles with favorable outcomes have a statistically significant higher citation velocity than articles without favorable outcomes (*P* = .018). Articles with favorable results had higher mean and median citation velocities (5.9 and 4.5) than those without favorable results (4.9 and 3.8). This represents an 18% increase in median citations per year for articles with favorable results.

Linear regression models were employed to determine significant predictors for both citation velocity and AAS. For citation velocity, the R-squared (adjusted) was 3.20%, showing very low explanation of variance, but all variables in the model were statistically significant (intervention category (*P* = .005), presence of industry funding (*P* = .031), and study result (*P* = .008). Outpatient-centered care studies (*P* = .006), surgical procedures and techniques studies (*P* = .003), and pain and perioperative management studies (*P* = .049) all had significant higher citation velocity than surgical technology studies. Studies with industry funding (*P* = .021) had significantly higher citation velocity compared to studies without industry funding. For AAS, the R-squared (adjusted) was 0.54%, and no variables were statistically significant. See Table 5 for full results.

**Table 5:** Multiple linear regression results.

Variable	Coef	95% CI	*P*-value
Citation velocity			
Constant	−0.344	(−0.544, −0.145)	.001
Intervention category			
Outpatient-centered care	0.482	(0.139, 0.824)	.006
Pain and perioperative management	0.212	(0.001, 0.424)	.049
Surgical procedures and techniques	0.356	(0.124, 0.588)	.003
Industry funding			
Undisclosed	−0.050	(−0.256, 0.157)	.636
Yes	0.250	(0.038, 0.463)	.021
Study results			
Favorable	0.225	(0.058, 0.392)	.008
AAS			
Constant	−0.115	(−0.317, 0.088)	.267
Intervention category			
Outpatient-centered care	0.142	(−0.205, 0.489)	.422
Pain and perioperative management	0.055	(−0.159,0.269)	.613
Surgical procedures and techniques	0.209	(−0.026, 0.444)	.081
Industry funding			
Undisclosed	−0.157	(−0.367, 0.052)	.141
Yes	0.109	(−0.106, 0.325)	.319
Study results			
Favorable	0.103	(−0.066, 0.273)	.231

CI, confidence interval.

## Discussion

This study identified an unequal distribution of impact among hip and knee arthroplasty RCTs published over the past decade, with a small subset of studies accounting for a disproportionately large share of both citations and online attention. Although randomized controlled trials remain essential for advancing evidence-based arthroplasty care, these findings suggest that not all RCTs contribute equally to the scientific discourse. Analyzing the distribution of citation velocity and Altmetric Attention Score (AAS) via the Gini coefficient reveals high degrees of impact concentrated among few RCTs. While citation data were moderately unequally distributed (Gini = 0.42), the distribution of AAS was significantly more concentrated (Gini = 0.77). This highlights the difference in dissemination between academic and public spheres and underscores the importance of examining the characteristics that may influence how RCTs are received, disseminated, and cited within arthroplasty research.

These results are consistent with prior bibliometric analyses across multiple medical specialties, which have demonstrated that citation distributions are skewed, with many articles receiving few or no citations at all [[38], [39], [40]]. Similar patterns have been observed within orthopedic literature, where analysis across subspecialties has shown that a substantial proportion of published articles accrue 5 or fewer citations over time [41]. Prior bibliometric orthopedic research looking at AAS has also noted a small subset of articles garnering disproportionate online attention [10]. To our knowledge, this is the first study in arthroplasty research to define the concentration of scholarly impact using the Gini coefficient. By applying this continuous measure to citation velocity and AAS, the present analysis moves beyond prior bibliometric approaches that relied on binary classifications of high- vs low-impact studies. This framework enables a definitive, quantitative assessment of the extent to which scholarly impact is concentrated among a small subset of RCTs.

To assess whether associations with impact persisted after accounting for multiple study characteristics, we performed multivariable linear regression. Although a few variables were statistically significant for citation velocity, the model showed low explanation of variance, and no variables were significant for AAS. Consequently, our primary conclusions regarding study characteristics and impact are based on the univariate analyses.

A key finding of this analysis was the association between statistically significant results and higher citation velocity. RCTs with favorable outcomes, defined as statistically significant findings in support of the experimental intervention, demonstrated an 18% increase in citation velocity compared to trials without favorable results. This pattern is consistent with prior findings across clinical research, where significant results have been shown to increase the likelihood of citation [[42], [43], [44]]. To our knowledge, this has not been demonstrated previously in arthroplasty research, and this may affect the visibility of certain RCTs found to have a “favorable” result, even if the clinical benefit is marginal or context dependent. The heightened visibility of favorable findings reinforces the importance of interpreting RCT results with careful attention to clinical significance and trial quality. As an independent variable, industry funding was not associated with either citation velocity or AAS. This is a reassuring finding, particularly in light of ongoing concerns about the influence of commercial interests on study design, interpretation, and dissemination [[45], [46], [47]]. This finding contributes to an evolving understanding of how impact is related to arthroplasty research quality, where prior work has found positive associations between AAS and methodological rigor [48].

The intervention category of the RCTs also demonstrated some association with AAS impact. Pain and perioperative management trials and those focused on outpatient-centered care received higher median AAS compared to those studying surgical technology. Given that Altmetric Attention Score reflects engagement from news media, social platforms, and the general public, as well as clinicians, interventions focused on patient-centered or postoperative concerns may be more likely to attract online attention. Nonetheless, with only 7% of all RCTs in the sample focused on outpatient-centered care, this presents an opportunity for future RCTs.

The weak correlation between citation velocity and AAS (r = 0.30) further highlights the distinct dimensions of article impact, and the correlation coefficient measured in this study is consistent with other reported values among orthopedic research [19,49]. While citations accumulate gradually and reflect academic engagement, AAS captures immediate attention across digital platforms, often peaking near the time of publication [19]. As such, high AAS does not necessarily predict sustained scholarly influence, and vice versa. Together, these metrics offer a complementary understanding of how research disseminates both professional and public spheres [11,19,50].

### Trends from the most impactful RCTs

While not designed for formal statistical comparison, examining the most impactful RCTs provides additional insight into arthroplasty research trends. Tables 1 and 2 highlight RCTs with Z-scores >3.00 for both AAS and citation velocity, respectively. Of the top 10 most cited studies, 8 focused on knee arthroplasty. Of note, 3 of those trials compared kinematic and mechanical alignment, and 2 looked at robotic assisted total knee arthroplasty. Similarly, 6 of the top 8 studies with the highest AAS scores centered on knee arthroplasty. In particular, Bell et al’s study on robotic-assisted total knee arthroplasty appeared on both lists and achieved the highest AAS overall, underscoring the past decade’s focus on robotic-assisted arthroplasty [21]. Together, these findings suggest that knee arthroplasty, particularly studies evaluating surgical technique and innovation, predominate in the upper tier of RCT influence in both academic and public domains. This observation is particularly notable given that formal statistical analyses of the sample demonstrated lower AAS scores for surgical technology RCTs compared with those addressing outpatient care or perioperative pain management. The prominence of surgical technology trials among the most influential studies, therefore, highlights a divergence between overall sample-wide trends and the subset of highly impactful papers, suggesting that select topics—such as robotic-assisted navigation—can transcend broader patterns and disproportionately shape conversation in the public sphere.

### Potential limitations

This study has several limitations. First, the classification of study outcomes as either favorable or not favorable was based exclusively on statistical significance in the primary outcome, without consideration of clinical relevance or the authors’ own interpretation of the findings. While this approach allowed for a more objective and reproducible analysis, it may not fully capture the nuanced judgments researchers and clinicians make when evaluating the practical implications of a study. Another limitation of this study concerns the classification of RCT interventions. While grouping trials by intervention type was a practical approach for statistical analysis, the 4 categories applied here were constructed in a somewhat subjective manner. In the absence of a formal framework or widely accepted standard, categorization was guided by consensus judgment of the article reviewers.

In terms of impact data, although citation velocity was used to normalize for time since publication, it remains an imperfect adjustment, as citation patterns can vary significantly by topic and may be influenced by external factors such as publication timing, editorial promotion, or inclusion in guidelines. Additionally, impact metrics such as Altmetric Attention Score are inherently dynamic and may fluctuate over time. Articles published closer to the data collection date may still have had less opportunity to accumulate citations or online attention. Moreover, AAS is driven by online engagement and the degree to which online attention in general has increased in recent years remains undetermined. Finally, although we examined several study characteristics associated with impact, there are likely other unmeasured factors influencing dissemination, such as author reputation, geographic trends, and institutional affiliations.

## Conclusions

Hip and knee arthroplasty RCTs published over the past decade demonstrate highly unequal patterns of impact and attention, with a small subset of trials accounting for most citations and online attention. Favorable results were associated with higher citation velocity, potentially indicating a citation bias that may amplify the perceived efficacy of certain interventions. The upper tier of RCTs published within the past decade tended to be focused on surgical technology and techniques related to knee arthroplasty. However, on average, RCTs focused on pain management and outpatient care generated greater online attention compared to those on surgical technology. These findings underscore the importance of considering both academic and public dissemination metrics, as citation velocity and AAS capture complementary but distinct dimensions of article impact.

## CRediT authorship contribution statement

**James R. Temple:** Writing – review & editing, Writing – original draft, Methodology, Investigation, Formal analysis, Data curation, Conceptualization. **Nadim Barakat:** Project administration, Methodology, Formal analysis, Data curation, Conceptualization. **Logan S. Carpenter:** Writing – review & editing, Investigation, Data curation. **Wendy M. Novicoff:** Writing – review & editing, Supervision, Methodology, Data curation, Conceptualization. **James A. Browne:** Writing – review & editing, Writing – original draft, Methodology, Conceptualization.

## Conflicts of interest

J. A. Browne reports receiving intellectual property royalties from OsteoRemedies, Enovis, and publishing royalties from Elsevier. He serves as a paid consultant for OsteoRemedies and Enovis. He holds stock in ForCast and stock options in Radlink. He serves in leadership roles as President of the Southern Orthopedic Association, as a Board Member of the Knee Society, and as a Steering Committee Member for the American Academy of Orthopedic Surgeons/American Joint Replacement Registry. He also serves as Associate Editor for the *Journal of Arthroplasty* and as Chair of the Miller Review Course for the *Journal of Bone and Joint Surgery*; all other authors declare no potential conflicts of interest.

For full disclosure statements refer to https://doi.org/10.1016/j.artd.2026.102046.

## Appendix A Supplementary data

Conflict of Interest Statement for Browne

Conflict of Interest Statement for Temple

Conflict of Interest Statement for Carpenter

Conflict of Interest Statement for Barakat

Conflict of Interest Statement for Novicoff

## References

[1] Bederman S.S., Wright J.G. (2012). Randomized trials in surgery: how far have we come?. J Bone Joint Surg Am.

[2] Chen Z., Bains S.S., Hameed D., Dubin J.A., Stern J.M., Mont M.A. (2022). Robust randomized controlled data is lacking in total joint arthroplasty. J Knee Surg.

[3] Ahmad S.S., Evangelopoulos D.S., Abbasian M., Röder C., Kohl S. (2014). The hundred most-cited publications in orthopaedic knee research. J Bone Joint Surg Am.

[4] Frazer P.M., Pastore G., McGarry A.K., Walsh T.P., Platt S.R. (2021). A bibliometric analysis of 4 major foot and ankle surgery journals. J Foot Ankle Surg.

[5] Movassagi K., Kunze K.N., Beck E.C., Fu M.C., Nho S.J. (2019). Predictors of 5-Year citation rate in the orthopaedic sports medicine literature. Am J Sports Med.

[6] Scarlat M.M., Mavrogenis A.F., Pećina M., Niculescu M. (2015). Impact and alternative metrics for medical publishing: our experience with International Orthopaedics. Int Orthop.

[7] Grover S., Elwood A.D., Patel J.M., Ananth C.V., Brandt J.S. (2022). Altmetric and bibliometric analysis of obstetrics and gynecology research: influence of public engagement on citation potential. Am J Obstet Gynecol.

[8] Gamble J.M., Traynor R.L., Gruzd A., Mai P., Dormuth C.R., Sketris I.S. (2020). Measuring the impact of pharmacoepidemiologic research using altmetrics: a case study of a CNODES drug-safety article. Pharmacoepidemiol Drug Saf.

[9] Kolahi J., Khazaei S., Iranmanesh P., Kim J., Bang H., Khademi A. (2021). Meta-analysis of correlations between altmetric attention score and citations in health sciences. Biomed Res Int.

[10] Tornberg H N., Cohen J.S., Gu A., Wei C., Mortman R., Sculco P.K. (2023). Impact of large database studies on orthopedic surgery literature: are we advancing the field?. HSS J.

[11] Mirghaderi S.P., Baghdadi S., Salimi M., Shafiei S.H. (2022). Scientometric analysis of the top 50 most-cited joint arthroplasty papers: traditional vs altmetric measures. Arthroplasty Today.

[12] Bala M.M., Akl E.A., Sun X., Bassler D., Mertz D., Mejza F. (2013). Randomized trials published in higher vs. lower impact journals differ in design, conduct, and analysis. J Clin Epidemiol.

[13] Sagliocca L., De Masi S., Ferrigno L., Mele A., Traversa G. (2013). A pragmatic strategy for the review of clinical evidence. J Eval Clin Pract.

[14] Elmore S.A. (2018). The altmetric attention score: what does it mean and why should I care?. Toxicol Pathol.

[15] Bernstein M., Feijoo E., Tate H., Pottayil F., Shah A. (2025). The 100 most impactful articles in foot and ankle surgery: an altmetric analysis. J Foot Ankle Surg.

[16] Kunze K.N., Polce E.M., Vadhera A., Williams B.T., Nwachukwu B.U., Nho S.J. (2020). What is the predictive ability and academic impact of the altmetrics score and social media attention?. Am J Sports Med.

[17] Ibrahim M.T., Imran H., Shuja M.H., Sheraz H., Howard A., Noordin S. (2024). Bibliometric analysis of predictors of altmetric attention scores in orthopedic research: investigating online visibility. Orthopedics.

[18] Burnham J.F. (2006). Scopus database: a review. Biomed Digit Libr.

[19] Collins C.S., Singh N.P., Ananthasekar S., Boyd C.J., Brabston E., King T.W. (2021). The correlation between altmetric score and traditional bibliometrics in orthopaedic literature. J Surg Res.

[20] Hasell J. (2023). “Measuring inequality: what is the Gini coefficient?” Published online at OurWorldinData.org. https://ourworldindata.org/what-is-the-gini-coefficient.

[21] Bell S.W., Anthony I., Jones B., MacLean A., Rowe P., Blyth M. (2016). Improved accuracy of component positioning with robotic-assisted unicompartmental knee arthroplasty: data from a prospective, randomized controlled study. J Bone Joint Surg Am.

[22] Nam D., Lawrie C.M., Salih R., Nahhas C.R., Barrack R.L., Nunley R.M. (2019). Cemented versus cementless total knee arthroplasty of the same modern design: a prospective, randomized trial. J Bone Joint Surg Am.

[23] Brozovich A.A., Incavo S.J., Lambert B.S., Sullivan T.C., Wininger A.E., Clyburn T.A. (2022). Intraosseous morphine decreases postoperative pain and pain medication use in total knee arthroplasty: a double-blind, randomized controlled trial. J Arthroplasty.

[24] Pongcharoen B., Liengwattanakol P., Boontanapibul K. (2023). Comparison of functional recovery between unicompartmental and total knee arthroplasty: a randomized controlled trial. J Bone Joint Surg Am.

[25] Wall P.D.H., Parsons N.R., Parsons H., Achten J., Balasubramanian S., Thompson P. (2017). A pragmatic randomised controlled trial comparing the efficacy of a femoral nerve block and periarticular infiltration for early pain relief following total knee arthroplasty. Bone Jt J.

[26] Tol M.C.J.M., van den Bekerom M.P.J., Sierevelt I.N., Hilverdink E.F., Raaymakers E.L.F.B., Goslings J.C. (2017). Hemiarthroplasty or total hip arthroplasty for the treatment of a displaced intracapsular fracture in active elderly patients: 12-year follow-up of randomised trial. Bone Jt J.

[27] Mont M.A., Beaver W.B., Dysart S.H., Barrington J.W., Del Gaizo D.J. (2018). Local infiltration analgesia with liposomal bupivacaine improves pain scores and reduces opioid use after total knee arthroplasty: results of a randomized controlled trial. J Arthroplasty.

[28] Sims A.L., Parsons N., Achten J., Griffin X.L., Costa M.L., Reed M.R. (2018). A randomized controlled trial comparing the Thompson hemiarthroplasty with the Exeter polished tapered stem and unitrax modular head in the treatment of displaced intracapsular fractures of the hip: the WHiTE 3: HEMI trial. Bone Jt J.

[29] Prvu Bettger J., Green C.L., Holmes D.N., Chokshi A., Mather R.C., Hoch B.T. (2020). Effects of virtual exercise rehabilitation In-Home therapy compared with traditional care after total knee arthroplasty: VERITAS, a randomized controlled trial. J Bone Joint Surg Am.

[30] Dossett H.G., Estrada N.A., Swartz G.J., LeFevre G.W., Kwasman B.G. (2014). A randomised controlled trial of kinematically and mechanically aligned total knee replacements: two-year clinical results. Bone Jt J.

[31] Kim Y.-H., Yoon S.-H., Park J.-W. (2020). Does robotic-assisted TKA result in better outcome scores or long-term survivorship than conventional TKA? A randomized, controlled trial. Clin Orthop.

[32] MacDessi S.J., Griffiths-Jones W., Chen D.B., Griffiths-Jones S., Wood J.A., Diwan A.D. (2020). Restoring the constitutional alignment with a restrictive kinematic protocol improves quantitative soft-tissue balance in total knee arthroplasty: a randomized controlled trial. Bone Jt J.

[33] Goyal N., Chen A.F., Padgett S.E., Tan T.L., Kheir M.M., Hopper R.H. (2017). Otto aufranc award: a multicenter, randomized study of outpatient versus inpatient total hip arthroplasty. Clin Orthop.

[34] Young S.W., Walker M.L., Bayan A., Briant-Evans T., Pavlou P., Farrington B. (2017). The chitranjan S. Ranawat award : no difference in 2-year functional outcomes using kinematic versus mechanical alignment in TKA: a randomized controlled clinical trial. Clin Orthop.

[35] Moffet H., Tousignant M., Nadeau S., Mérette C., Boissy P., Corriveau H. (2015). In-Home telerehabilitation compared with face-to-face rehabilitation after total knee arthroplasty: a noninferiority randomized controlled trial. J Bone Joint Surg Am.

[36] Lamplot J.D., Wagner E.R., Manning D.W. (2014). Multimodal pain management in total knee arthroplasty: a prospective randomized controlled trial. J Arthroplasty.

[37] Cheng T.E., Wallis J.A., Taylor N.F., Holden C.T., Marks P., Smith C.L. (2017). A prospective randomized clinical trial in total hip arthroplasty-comparing early results between the direct anterior approach and the posterior approach. J Arthroplasty.

[38] Callaham M., Wears R.L., Weber E. (2002). Journal prestige, publication bias, and other characteristics associated with citation of published studies in peer-reviewed journals. JAMA.

[39] Abdalla M., Abdalla S., Abdalla M. (2023). Tracing the path of 37,050 studies into practice across 18 specialties of the 2.4 million published between 2011 and 2020. Elife.

[40] Ranasinghe I., Shojaee A., Bikdeli B., Gupta A., Chen R., Ross J.S. (2015). Poorly cited articles in peer-reviewed cardiovascular journals from 1997 to 2007: analysis of 5-year citation rates. Circulation.

[41] Kortlever J.T.P., Tran T.T.H., Ring D., Menendez M.E. (2019). The growth of poorly cited articles in peer-reviewed orthopaedic journals. Clin Orthop.

[42] Duyx B., Urlings M.J.E., Swaen G.M.H., Bouter L.M., Zeegers M.P. (2017). Scientific citations favor positive results: a systematic review and meta-analysis. J Clin Epidemiol.

[43] Jannot A.-S., Agoritsas T., Gayet-Ageron A., Perneger T.V. (2013). Citation bias favoring statistically significant studies was present in medical research. J Clin Epidemiol.

[44] Boyd C.J., Gentry Z.L., Martin K.D., Rais-Bahrami S. (2019). Factors associated with the highest and lowest cited research articles in urology journals. Urology.

[45] Haislup B.D., Gupta S., Fleisher I., Murthi A.M., Wright M.A. (2024). Funding bias in shoulder arthroplasty research. J Shoulder Elbow Surg.

[46] Okike K., Kocher M.S., Mehlman C.T., Bhandari M. (2007). Conflict of interest in orthopaedic research. An association between findings and funding in scientific presentations. J Bone Joint Surg Am.

[47] Leopold S.S., Warme W.J., Fritz Braunlich E., Shott S. (2003). Association between funding source and study outcome in orthopaedic research. Clin Orthop.

[48] Kunze K.N., Richardson M., Bernstein D.N., Premkumar A., Piuzzi N.S., McLawhorn A.S. (2020). Altmetrics attention scores for randomized controlled trials in total joint arthroplasty are reflective of high scientific quality: an altmetrics-based methodological quality and bias analysis. J Am Acad Orthop Surg Glob Res Rev.

[49] Kunze K.N., Vadhera A.S., Polce E.M., Higuera C.A., Siddiqi A., Chahla J. (2023). The altmetric attention score is associated with citation rates and may reflect academic impact in the total joint arthroplasty literature. HSS J.

[50] Shiah E., Heiman A.J., Ricci J.A. (2020). Analysis of alternative metrics of research impact: a correlation comparison between altmetric attention scores and traditional bibliometrics among plastic surgery research. Plast Reconstr Surg.

