# Supplementary material for: Citation Velocity and Social Media Impact Analysis of Hip and Knee Arthroplasty Randomized Controlled Trials
Source: Arthroplast Today. 2026 May 17;39:102046. doi: 10.1016/j.artd.2026.102046 (PMC13197701; doi:10.1016/j.artd.2026.102046)
Supplement: Conflict of Interest Statement for Browne [file mmc1.docx]

# CONFLICT OF INTEREST STATEMENT

***American Association of Hip and Knee Surgeons***

(Adopted from the American Academy of Orthopaedic Surgeons disclosure statement)

The following form **must be filled out completely and submitted by each author (example, 6 authors, 6 forms).**

**All items require a response. If there is no relevant disclosure for a given item, enter "*None*.”**

Citation Velocity and Social Media Impact Analysis of Hip and Knee Arthroplasty Randomized Controlled Trials

1. Royalties from a company or supplier (The following conflicts were disclosed)

OsteoRemedies – IP royalty payments to author

Enovis – IP royalty payments to author

2. Speakers bureau/paid presentations for a company or supplier (The following conflicts were disclosed)

None

3A. Paid employee for a company or supplier (The following conflicts were disclosed)

None

3B. Paid consultant for a company or supplier (The following conflicts were disclosed)

OsteoRemedies – Consulting fees paid to author

Enovis – Consulting fees paid to author

3C. Unpaid consultants for a company or supplier (The following conflicts were disclosed)

None

4. Stock or stock options in a company or supplier (The following conflicts were disclosed)

ForCast – Stock ownership

Radlink – Stock options

5. Research support from a company or supplier as a Principal Investigator (The following conflicts were disclosed)

None

6. Other financial or material support from a company or supplier (The following conflicts were disclosed)

None

7. Royalties, financial or material support from publishers (The following conflicts were disclosed)

Elsevier – Publishing royalties paid to author

8. Medical/Orthopaedic publications editorial/governing board (The following conflicts were disclosed)

Journal of Arthroplasty – Associate Editor

Journal of Bone and Joint Surgery – Chair, Miller Review Course

9. Board member/committee appointments for a society (The following conflicts were disclosed)

Southern Orthopaedic Association – President

The Knee Society – Board Member

AAOS/AJRR – Steering Committee Member

**Each author must sign AND print or type his/her name, date and submit a separate form**

In addition, one BLINDED Conflict of Interest form (no author names used) should be submitted per manuscript with all author disclosures.

James A. Browne James A. Browne 2/5/2026

Author Name (Print or Type) Author Signature Date
